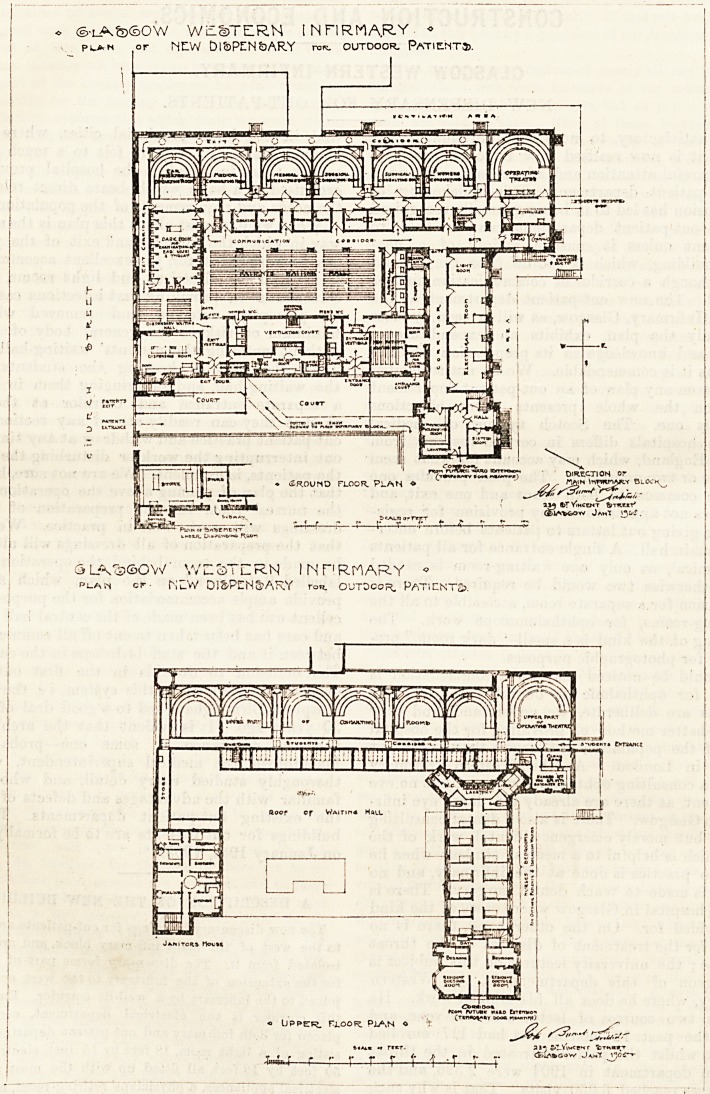# Glasgow Western Infirmary

**Published:** 1905-01-21

**Authors:** 


### HOSPITAL ADMINISTRATION.

### CONSTRUCTION AND ECONOMICS.

### GLASGOW WESTERN INFIRMARY.

NEW DISPENSARY FOR OUT-PATIENTS.

It is satisfactory to note that throughout the country it is now realised how important it is to expend careful attention and adequate thought upon the out-patient department. This quickening of apprehension has led to it being generally recognised that no out-patient department can be held to be efficient unless it consists mainly of a one-storied building, which is cut off from the hospital proper, though a corridor of communication may be provided. The new out-patient department at the Western Infirmary, Glasgow, as will be seen by those who study the plan, exhibits an expenditure of thought and knowledge in its preparation which is as rare as it is commendable. We do not remember to have seen any plan of an out-patient department which on the whole presents fewer objections than this one. The Scotch system of medical relief at hospitals differs in certain respects from those in England, which may account for what seem to be one or two omissions. The plan provides one entrance common to both sexes and one exit, and there does not appear to be any provision for registering or giving out letters to patients before entering the main hall. A single entrance for all patients is economical, as only one waiting-room is needed where otherwise two would be required. There is no provision for a separate room, accessible to all the consulting-rooms, for ophthalmoscope work. The only thing of the kind is a small "dark room," presumably for photographic purposes.

It should be noticed that no accommodation is afforded for ophthalmic or dental cases. These omissions are deliberate, we understand, and they reveal a better method of providing for the hospital needs of the population in Glasgow than at present prevails in London. At the Western Infirmary there is a consulting ophthalmic surgeon, but no eye department, as there are already two large eye infirmaries in Glasgow. There is also a dental consulting surgeon, but merely emergency dental work of the kind which is helpful to a medical student when he goes into practice is done at the infirmary, and no attempt is made to teach dental surgery. There is a dental hospital in Glasgow where cases of the kind are provided for. On the other hand, there is no hospital for the treatment of diseases of the throat and nose; the university lecturer on this subject is the surgeon of this department in the Western Infirmary, where he does all his clinical work. He conducts two courses of lectures in the year, and during the past 12 months has had 117 enrolled students, whilst the patients treated in the throat and nose department in 1904 were 2,396, and the attendances reached 6,950 visits. That is why such excellent accommodation has been made in the following plans at the Western Infirmary, which will remove the need for a throat and ear hospital in Glasgow. We mention these facts as indicating that in the large provincial cities, where public opinion makes its weight felt to a much greater extent than in London, the hospital provision is organised on a basis which bears direct relation to the needs and requirements of the population.

What we like most about this plan is the masterly way in which the entrance and exit of the patients has been provided for, the excellent accommodation secured for the electrical and light rooms, and the care shown in providing that infectious cases shall be promptly detected and removed with the minimum of risk to the general body of patients without entering the patients' waiting-hall at all. Then the idea of keeping the students out of the waiting-hall, and of bringing them in through a separate entrance and corridor at the back, so that they can readily attend any section of the out-patient practice and withdraw at any time, without interrupting the work or disturbing the staff or the patients, is excellent. We are not sure, however, that the plan of placing above the operation-theatre the nurses' room for the preparation of surgical dressings will work well in practice. We expect that the preparation of all dressings will ultimately be made in the room behind the operation-theatre labelled "steriliser" on the plan, which seems to provide ample accommodation for the purpose. Excellent use has been made of the central hall method, and care has been taken to cut off all communication between it and the staff bedrooms in the east wing. The omission to do this in the first out-patient department planned on this system, *i.e.* the Queen's Hospital, Birmingham, led to a good deal of trouble 30 years ago. It is evident that the architect has had the assistance of some one—probably Dr. Mackintosh, the medical superintendent, who has thoroughly studied every detail, and who is also familiar with the advantages and defects of most of the existing out-patient departments. The new buildings for out-patients are to be formally opened on January 19th, 1905.

### A DESCRIPTION OF THE NEW BUILDINGS.

The new dispensary buildings for out-patients are situated to the west of the main infirmary block, and are entirely isolated from it. The dispensary forms part of a scheme for the extension of the infirmary to the west, and will be joined to the infirmary by a well-lit corridor. Entering off this corridor is the electrical department, conveniently placed for both infirmary and out-patient departments, consisting of a light room, 18 feet by 15 feet, electrical room, 50 feet by 18 feet, all fitted up with the most up-to-date electrical appliances, a physicians' retiring-room, and a suite of rooms on the first floor for the accommodation of nurses attached to this department.

The entrance to the dispensary proper is from Church Street, a street running north from Dumbarton Road, in which is situated the main entrance to the infirmary grounds. Entrance and exit gates are provided leading to courtyards separating the entrance and exit doorways.

The new patients' waiting-room is 26 feet by 16 feet, and is in communication with a diagnosis-room for the examination and classification of cases. The diagnosis-room is

Patients enter by the east doorway, and, under the control of the porter, are directed, on their first visit, into the waiting-room for new patients, where all inquiries are made, the old patients passing into the general waiting-hall.

lighted by a vertical and roof-light on the north side, and adjoining it is a well-lit room for the isolation of any suspicious or infectious case. This room has a separate door to an ambulance-court.

The general waiting-hall, 86 feet by 31 feet, is seated for over 400 persons; the patients are classified into groups for convenience of distribution into the various surgical and medical consulting-rooms; suitable male and female lavatory accommodation is provided in open courts adjoining the waiting-hall.

From the waiting-hall the communication corridor leads eastward, to the main operating theatre—30 feet by 29 feet—with gallery accommodation for 60 students. This room is lighted by vertical and roof-light from the north, and has attached to it a sterilising and a recovery-room, the latter divided for male and female patients, and, above this there is a nurses' room for the preparation and storage of surgical dressings, etc. Immediately to the east of the waiting-hall is the surgical dressing-room fitted with arm and foot sinks and divided by enamelled slate partitions into separate compartments.

Small lobbies lead off the waiting-hall to the medical and surgical consulting-rooms with their necessary dressing-rooms. These five consulting-rooms are 24 feet by 24 feet, each having gallery accommodation for 50 students. The lighting, as in the operating theatre, is from the north.

At the west end the communication-corridor leads into the Ear, Nose, and Throat Rooms, consisting of a consulting-room 30 feet by 24 feet, having gallery accommodation for 50 students (lighted as before), and communicating by a sliding door with a dark room, 37 feet by 24 feet, fitted with 18 stalls for the examination of patients by artificial light.

From each of the consulting-rooms separate doors lead to the exit corridor, which runs along the entire north and west walls of the building and ends in the Dispensary waiting hall. This hall, 30 feet by 19 feet, is seated for 70 persons, and has two service openings, with sliding doors from the dispensing room, and adjoins the exit vestibule near the street.

The dispensing-room is 30 feet by 20 feet, and is furnished with all the most modern fittings and appliances. Adjoining this room is the laboratory, which contains the aerated water machine and an outfit of steam pans. Connected with the laboratory is a fireproof room for the storage of inflammables.

There is also a basement for the storage of surgical dressings, etc., with goods-hoist. A subway will eventually lead from this basement to the new west ward block.

The walls and floors have been carefully considered from a sanitary point of view. The floors are Terrazzo throughout, except in the waiting-hall where the patients are seated, the floors there being wood-block. The walls of the vestibules and waiting-halls are faced with terra-cotta and majolica; the consulting-rooms, dressing-rooms, and corridors being finished with glazed tiles, the whole being designed with a view to facilitate cleanliness.

Various devices have been employed for the easy and mechanical control of the patients and their passage through the building; the doctor in charge of each consulting-room being able to signal to the general waiting hall for a new patient as he passes the former one out into the corridor, which has lettered tiles directing to exit, thus preventing patients wandering over the building.

All basins, sinks, etc., have been specially designed to suit the requirements of such a building. The access door for students is on the first floor at the east side of the building, and from this a corridor, with cloak-room accommodation, leads directly to the upper level of the galleries in the various consulting-rooms, one stair only being provided at the west end of this corridor.

The buildings are heated on the "Reck" system, air being admitted by a series of ducts in the basement, and passing from them through inlet gratings situated behind the radiators, the foul air being extracted at the ceiling through shafts provided with electrical fans, the windows can also be opened if necessary.

Accommodation has been provided for the janitor over the dispensary-room and waiting hall in the south-west corner.

The building has been constructed from plans prepared by John James Burnet, A.R.S.A.

## Figures and Tables

**Figure f1:**